# Design of an adiabatic T2-preparation method optimized for cardiac motion and flow insensitivity at 3T

**DOI:** 10.1186/1532-429X-13-S1-P2

**Published:** 2011-02-02

**Authors:** Elizabeth Jenista, Wolfgang Rehwald, Igor Klem, Michele Parker, Enn Ling Chen, Raymond Kim

**Affiliations:** 1 Duke University, Durham, NC, USA; 2 Siemens, Chicago, IL, USA

## Objective

To develop a T_2_-prep method for cardiac imaging at 3T which is less sensitive toward B_1_ inhomogeneity, flow and motion than previously described techniques.

## Background

T_2_ contrast is important for the assessment of acute myocardial edema and coronary morphology. At 1.5T, the standard T_2_-prep method uses 4 composite 180° pulses (MLEV4[^1^]) providing robust and uniform T_2_-preparation, but at 3T, increased B_1_ inhomogeneity causes artifacts. Decreasing inter-pulse spacing improves refocusing in the presence of motion and flow. We developed a configurable T_2_-preparation allowing up to 4 adiabatic refocusing pulses (B_1_-insensitive) to study the effect of inter-pulse spacing on motion-robustness. We compared image homogeneity using MLEV4 as reference.

## Methods

The modules employed 800 µs rectangular tip-down /flip-back pulses and a series of adiabatic refocusing pulses (BIREF-1[^2^]). With these modules, we acquired mid-ventricular short axis cardiac images in healthy volunteers at 3T (MAGNETOM Verio, Siemens) using an ECG-gated, TurboFLASH sequence and different T_2_-prep times ranging from ≤35 ms to 120 ms, in systole and diastole. Four modules were used: A1 (1xBIREF-1), A2 (2xBIREF-1), A4 (4xBIREF-1), and MLEV 4. Separate contours were drawn for myocardium and cavity using ImageJ (NIH). The coefficient of variation was used as a measure of inhomogeneity. Readers (n=3) blinded to the T_2_-preparation scored images on a four-point scale for myocardial/cavity inhomogeneity and endocardial border definition. Statistical comparisons were made by ANOVA with Bonferroni correction.

## Results

**Figure 1:**
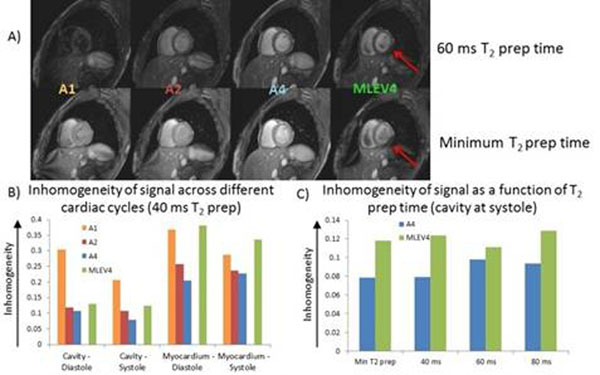
Figure 1a shows representative images from each sequence at 60 ms (top) and at the minimum T_2_-prep time (bottom). Defects in the myocardium from the MLEV4 sequence are highlighted with red arrows. 1b) This graph shows the effect of the number of refocusing pulses on signal homogeneity. Increasing the number of pulses decreases the inhomogeneity. 1c) The adiabatic refocusing pulses in A4 provide improved image homogeneity compared with the composite pulses in the MLEV4 sequence.

The effect of the number of pulses and inter-pulse delay is demonstrated in figure 1a and 1b. Signal and homogeneity of myocardium and cavity increased with the number of pulses. The inhomogeneity of the cavity in diastole as measure of flow sensitivity (T_2_-prep time 40 ms) decreased with increasing pulse number (A1 = 0.3, A2 = 0.12, A4 = 0.11 and MLEV4 = 0.13). A comparison of A4 with MLEV4 (figure 1c) highlights the improvement due to adiabatic pulses. MLEV4 and A4 have similar inter-pulse spacing, yet across all T_2_-prep times, A4 performed better. From the visual scores, A4 scored higher and was ranked first among all sequences.

## Conclusions

We developed a B_1_, motion and flow insensitive T_2_-prep method using adiabatic pulses. Using BIREF-1 refocusing pulses allows shorter inter-pulse spacing without exceeding SAR limitations, and improves homogeneity over the MLEV4 composite pulses.

**Table 1:** Results from visual scoring of images. * = statistically significant (P<0.05) vs MLEV4

	A1	A2	A4	MLEV4
Mean Quality Score	0.17	1.33	2.42	1.83
Mean Rank	0	1.25	2.75*	2.00
